# Team-based learning versus interactive lecture in achieving learning outcomes and improving clinical reasoning skills: a randomized crossover study

**DOI:** 10.1186/s12909-022-03411-w

**Published:** 2022-05-07

**Authors:** Muhammad Imran, Taher Fawzy Halawa, Mukhtiar Baig, Ahmed Mohammed Almanjoumi, Mohammed Mustafa Badri, Waleed Ahmed Alghamdi

**Affiliations:** 1 Department of Surgery, Medical Education Unit, Faculty of Medicine in Rabigh, King Abdulaziz University, Building 13, PO Box No. 80200, Jeddah, 21589 Saudi Arabia; 2 Department of Paediatrics, Faculty of Medicine in Rabigh, King Abdulaziz University, Jeddah, Saudi Arabia; 3 Department of Clinical Biochemistry, Assessment Unit, Faculty of Medicine in Rabigh, King Abdulaziz University, Jeddah, Saudi Arabia; 4 Department of Surgery, Faculty of Medicine in Rabigh, King Abdulaziz University, Jeddah, Saudi Arabia; 5 Division of Psychiatry, Faculty of Medicine, King Abdulaziz University, Department of Medicine, Faculty of Medicine in Rabigh, King Abdulaziz University, Jeddah, Western Region Saudi Arabia

**Keywords:** Team-based learning, Interactive lecture, Clinical reasoning skills, Learning outcomes

## Abstract

**Background:**

This study aimed to investigate the impact of interactive lecture (IL) and team-based learning (TBL) on improving clinical reasoning skills (CRSs) and achieving learning outcomes (LO). Students’ feedback was obtained about the strategies.

**Methods:**

This study was carried out at the Faculty of Medicine in Rabigh, King Abdulaziz University, Jeddah, Saudi Arabia. Two modules, endocrinology, and emergency were selected. Students of each batch in both modules were divided into two arms. With a randomized crossover design, IL & TBL were used for two separate topics in each module. After each topic, a quiz in the form of well-structured MCQs was taken. A questionnaire was designed to obtain students’ feedback. SPSS version 23 was used to analyse results. The difference between the mean values was calculated by Student’s t-test. Feedback data is presented as frequency. *P*-value ≤ 0.05 was considered statistically significant.

**Results:**

Learning outcomes were achieved by all groups in two modules, with both instructional strategies, IL and TBL. Students attempted >70% correct answers. However, in the emergency module, the groups with TBL as the instructional strategy performed better in quiz1 and quiz 2 (*p* = 0.026 and *p* = 0.016, respectively). Similarly, in the endocrinology module (3^rd^ year), although the groups with TBL as the instructional strategy performed better in both quizzes, it was significant in quiz1 (*p* = 0.02). The difficulty indices of the clinical reasoning questions (CRQ) were used as the parameters for comparison. In the emergency module, group1, in quiz1, with TBL as an instructional strategy performed better in the CRQ (*p* = 0.017), while in quiz2, group2 with TBL as the instructional strategy performed better (*p* < 0.001). Group1 of the third-year students (endocrinology module) performed better in the CRQ in quiz 1 with TBL as an instructional strategy than group 2 with IL (*p* = 0.04). Mostly, students in both modules preferred TBL over IL, and especially they liked team application. Students perceived that TBL was a better strategy to learn CRS.

**Conclusions:**

Students achieved LOs and CRS better with TBL as an instructional strategy. They preferred TBL over IL. It is suggested to include TBL, or increase its percentage, in the curriculum.

## Background

Lecture has been used for a long time in medical education [1]. Due to concerns over the one-way passage of knowledge and the passive role of learners, there is an emphasis on making lectures interactive. It has been argued that interaction during a lecture enhances learners’ attention, and participants usually appreciate interactive sessions [2]. Interaction varies, but the core purpose is to engage students in the learning process. Different strategies have been proposed to make lectures more interactive. Interactions are proposed keeping in mind adult learning theories [3]. Lecture is still trendy in our institute due to many factors, and interactive lecture (IL) is popular among faculty members.

There are several methods in small group discussions, including problem-based learning (PBL) and case-based learning (CBL), among others. These strategies have been supported by literature [4, 5]. However, instructors face many challenges while using PBL or CBL. Feasibility issues, the number of faculty members required to conduct small group discussions, and reliability among groups regarding achieving outcomes can be demanding. Team-based learning (TBL) has been introduced to tackle these issues, as one instructor can conduct it even for a class of more than a hundred students [6]. TBL is a relatively new but well-established instructional method in medical education. It is an active learning instructional strategy based on constructivist learning theory. Its main pillars include a focus on the learner instead of a teacher, the teacher’s role as a facilitator, constructing knowledge based on previous knowledge, interaction with other learners, and reflection [7].

There is an emphasis on competency-based education (CBE) in higher education for the last many years [8], and competency-based medical education (CBME) has gained popularity in medical schools [9]. Certain predefined abilities are incorporated as outcomes in a curriculum, and the whole curriculum is then organized according to those outcomes or competencies [10]. In Saudi Arabia, there is a move toward CBME, and the Saudi Medical Education Directives (Saudi-MED) framework has been developed to meet these issues. Certain competencies, or sub-competencies, which a graduate doctor must achieve, are critical thinking, clinical reasoning skills (CRS), collaboration, self-learning, and teamwork [11]. CRS is imperative in medical education. It has been emphasized that throughout the medical school years, clinical reasoning should be included in the curriculum. These measures can increase the diagnostic accuracy of doctors during their practice [12].

The faculty of medicine in Rabigh (FMR) is a relatively newly established faculty in Saudi Arabia [13]. This is the first time we introduced TBL as a teaching and learning strategy. Despite the importance of TBL in the healthcare profession, its use is limited in our local context. It is being used scarcely in our faculty, and a study from the sister faculty demonstrates the lack of students’ knowledge about TBL [14]. Due to the significance of TBL in the curriculum, it is imperative to compare it with another established strategy, IL, which is being used frequently in our institute. The main purpose of this study was to compare the impact of learning strategies, TBL and IL, on students’ learning outcomes and clinical reasoning skills (CRSs). The study’s findings would help design better teaching and learning strategies and bring changes in the curriculum. These findings can be helpful for other institutes as well. In this background, the present randomized crossover study was designed with the following objectives:

- to investigate the impact of instructional strategies, IL and TBL, on improving clinical reasoning skills and achieving learning outcomes in a basic (third year) and a clinical (sixth year) sciences module.
- to acquire students’ feedback regarding IL and TBL as instructional strategies.

## Methods

### Study design, settings, and ethics

The present randomized crossover study was carried out at the male campus of the faculty of medicine in Rabigh (FMR), King Abdulaziz University (KAU), Jeddah, Saudi Arabia, in the year 2019 out after seeking approval from the institute’s biomedical ethics research committee (reference no. 360–19). FMR aims at providing an active learning approach through an integrated, modular system [15]. The participants were informed, after which written consent was taken. Medical students in Saudi Arabia spend their first year (preparatory year) learning English, chemistry, biology, and other key premedical topics. The preclinical year starts from the 2nd year, and 6th year is considered the final year [16].

### Participants and modules

Two batches of students were included in the study, one from the 3^rd^ year and the other from the 6^th^ year. Two modules were chosen: the endocrinology module from the preclinical (3^rd^) year and the emergency module from the clinical (6^th^) year. The endocrinology module is an integrated module that includes sessions from basic and clinical sciences. It also covers in-class teaching, small group sessions, and practical sessions. Some sessions include input from many departments. Common emergency cases are discussed in the emergency module in different ways, including in-class sessions, clinical rotations, and practical sessions in the clinical skills lab. The modules were selected for different reasons. First, it was investigated whether there was any difference in our study outcomes according to the academic year’s level. Secondly, students’ feedback was required in an integrated module and a purely clinical module. Feasibility was another consideration in selecting these modules. The issue of randomization was discussed in detail with both batches of students, and they allowed the research team to randomly allocate students in two arms. Students of each batch in both modules were divided into two arms. There was a random allocation for each arm. We used a random numbers table to include each batch of students in two arms [17].

### Learning materials

Topics to be discussed were provided in the study guides before the modules started. Learning outcomes of the modules and learning objectives of the sessions were given in the study guides. Reference material was provided to students beforehand. Students were briefed about the instructional strategies being used for this study, and they were oriented about TBL.

### Teaching and learning strategies

Two different strategies, interactive lecture (IL) and team-based learning (TBL), were selected under the teaching and learning theme. It was made sure that students spent a certain amount of time for self-study, for both strategies, before they attended the in-class session. IL was defined as a teaching strategy in which the teacher delivered a lecture in an interactive way, i.e., with more students’ interaction during the session. Objectives were already given in the study guide. IL was delivered in the form of a lecture with a PowerPoint presentation. The in-class duration of IL was one hour (60 min) with two well-structured activities. One activity was in the form of brainstorming, in which students, in small groups, generated a list of issues or solutions in response to a critical question. The second activity was a small quiz. As the in-class duration of IL was less than TBL, two hours were allocated for self-study in the campus library. It was made sure that students would spend two hours in the library to read and discuss the critical points mentioned in the study guide. One demonstrator was responsible for monitoring their presence in the library.

TBL was conducted in a well-structured format as mentioned in the literature [18]. The total in-class duration of TBL was 85 min. There was time for self-preparation already mentioned in the study guide. Participants were divided into small teams comprising five to six members. Teams were formed by the instructor conducting TBL. In the class, the individual readiness assurance test (iRAT) was given in the form of 10 multiple-choice questions (MCQs), single best answer (SBA) type. The questions used in the TBL sessions were not repeated in any quiz. Time allocation was fifteen minutes, and each student solved those questions individually. Subsequently, the same test was conducted as a team readiness assurance test (tRAT). It was observed that the students solved the questions in teams—the given time was fifteen minutes. Thereafter, there was an instructor clarification review for fifteen minutes. The instructor clarified any point which was not clear to any team. This was followed by team application (tAPP). Different scenarios, with all relevant details, were provided to all teams. The rules were: 1. clinical scenarios had to be the same for all teams; 2. problems were significantly relevant to the students in their clinical practice; 3. there was a specific choice for each problem; 4. all teams would be required to display their answers simultaneously. Each team had to defend the answer. The duration of tAPP was 40 min.

### Execution of study

During the first week of each module, one arm of the participants in each group attended IL as an instructional strategy for the selected topic. The other arm attended TBL for the same topic. After finishing the topic, the assessment was conducted in the form of a quiz for both arms of students. In each module, the quiz comprised of fifteen MCQs, with the inclusion of clinical reasoning (higher cognitive level) questions. The emergency module questions were based on two outcomes: 1. discuss common emergency and trauma presentations, 2. develop a management plan for different emergency and trauma situations, and the questions of the endocrinology module were based on two outcomes: 1. describe the pathophysiology of endocrinal problems, 2. elucidate important investigations and treatment of endocrinology diseases. Following a discussion with two content experts and the research group, it was decided to consider the outcomes’ achievement if students attempted > 70% correct answers. MCQs were selected based on validity and reliability in the past exams. Questions with the difficulty index between 0.3 to 0.8 and the discrimination index ≥ 0.2 were selected. MCQs were discussed with two subject specialists and one member from the medical education unit. Similarly, another topic was selected for each module for the second week. This time, students were divided with crossover design, i.e., the group who received IL in the first week got TBL and vice versa. The second quiz was taken in the form of fifteen MCQs with all the aforementioned conditions, following which the results of both groups were compared.

### Statistical analysis

SPSS version 23 was used to analyse results. *P*-value ≤ 0.05 was considered statistically significant. The continuous data is mentioned as mean and standard deviation. The difference between the mean values of total marks acquired in quizzes by different strategies in both groups was calculated by Student’s t test. Comparison of difficulty indices of clinical reasoning questions in quizzes, with instructional strategies TBL and IL as a randomized crossover design, were calculated by applying Student’s t test. Feedback data is presented as frequency.

**Fig. 1:**
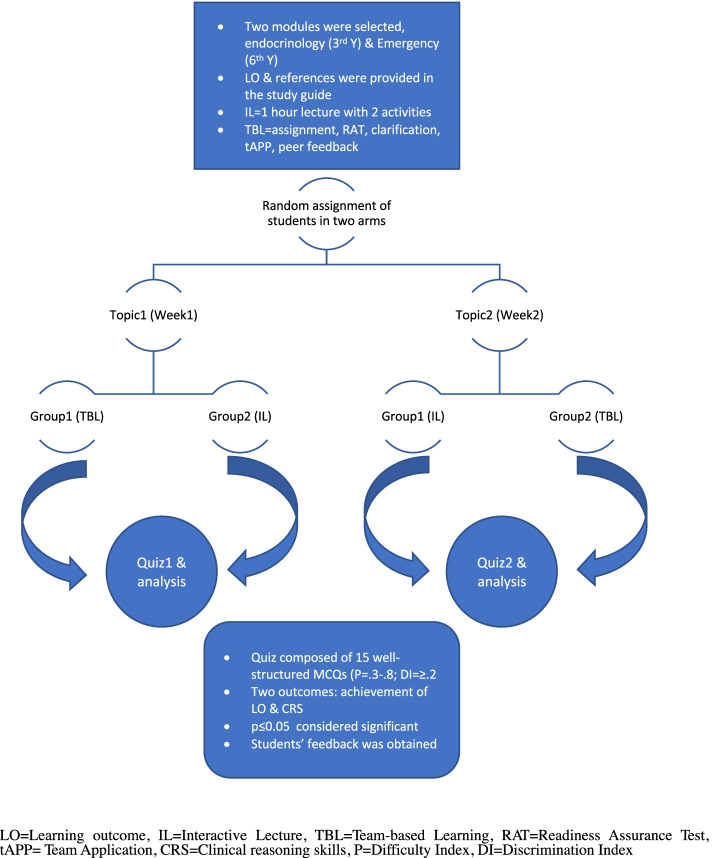
A brief summary of randomized crossover design

The methodology is summarized in Fig. 1.

## Results

Thirty-five male students were enrolled in the endocrinology module (3^rd^ year). The mean age was 21.9 (SD ± 1.5) years. Meanwhile, thirty-seven male students were enrolled in the emergency module (6^th^ year), with the mean age being 25.1 (SD ± 1.3) years.

Outcomes were achieved by all groups in two modules, with both instructional strategies, IL and TBL; students attempted more than seventy percent correct answers (Table1). However, in the emergency module, the groups with TBL as the instructional strategy performed better; the difference was statistically significant in quiz1 and quiz2, (*p* = 0.026 and *p* = 0.016, respectively) (Table1). Similarly, in the endocrinology module (3^rd^ year), although the groups with TBL as the instructional strategy performed better in both quizzes, it was significant in quiz1 (*p* = 0.02). In quiz 2, the results were not statistically significant (*p* = 0.132) (Table1).

**Table 1:** Comparison of marks in quizzes with instructional strategies TBL and IL as a randomized crossover design

Variables	Marks (percentage of total)	Marks (percentage of total)	*P*-value	95% Confidence interval (CI)	
				Lower band	Upper band
Emergency module (6^th^ year)	Group1 (*n* = 19)	Group2 (*n* = 18)			
Quiz1	88.77 ± 13.34^a^	77.84 ± 11.55^b^	0.026*	1.39	20.46
Quiz2	74.96 ± 11.63^b^	85.03 ± 11.09^a^	0.016*	2.01	18.18
Endocrinology module (3^rd^ year)	Group 1 *N* = 17	Group 2 *N* = 18			
Quiz1	85.40 ± 13.06^a^	76.48 ± 16.19^b^	0.02*	2.01	15.81
Quiz2	80.58 ± 12.67^b^	85.58 ± 12.67^a^	0.132	-1.45	10.83

^a^Instructional strategy TBL

^b^Instructional strategy IL

^*^statistically significant *p*-value

Group1 and group2 of the 6^th^ year students (emergency module) were compared, in quiz1 and quiz2, for answering the clinical reasoning questions (CRQ). The difficulty indices of the CRQ were used as the parameters for comparison. In quiz1, group1 with TBL as an instructional strategy performed better in the CRQ (*p* = 0.017), while in quiz2, group2 with TBL as the instructional strategy performed better (*p* < 0.001). Group 1 of the third-year students (endocrinology module) performed better in the CRQ in quiz 1 with TBL as an instructional strategy in comparison to group 2 with IL as an instructional strategy (*p* = 0.04). In Quiz 2, there was no statistical difference. Still, students with TBL as an instructional strategy performed better (Table 2).

**Table 2:** Comparison of difficulty indices of clinical reasoning questions in quizzes with Instructional strategies TBL and IL as a randomized crossover design

Sixth year (emergency module)				Third-year (endocrinology module)			
Variables	Mean difficulty index Group 1 (*N* = 19)	Mean difficulty index Group 2 (*N* = 18)	*P*-value	Variables	Mean difficulty index Group 1(*N* = 17)	Mean difficulty index Group 2 (*N* = 18)	*P*-value
Quiz 1	.81 ± .16^a^	.61 ± .18^b^	.017^#^	Quiz1	.76 ± .09^a^	.61 ± .11^b^	.04^#^
Quiz2	.63 ± .13^b^	.83 ± .11^a^	< .001^#^	Quiz2	.70 ± .14^b^	.78 ± .10^a^	.32

^a^ Instructional strategy TBL

^b^ Instructional strategy IL

^#^statistically significant *p*-value

The feedback provided by students is given in Table 3. Mostly, students enjoyed the interaction in IL and team application in TBL. They perceived that TBL was a better strategy to learn CRS (Table 3). Some open remarks of students about IL and TBL were interesting. One student wrote, “Lecture is an important mode of instruction, but I enjoy a lecture if it is a two-way process”. Another student remarked, “No more lectures please! I want discussion”. Similarly, a student stated the following about TBL, “iRAT followed by tRAT helps to correct our mistakes immediately. I feel the energy while working in a team”. Another student said, “Team application is the best part of TBL; it is challenging and interesting simultaneously. I felt like a working doctor.” Meanwhile, a student revealed, “Peer evaluation was the most interesting part for me. I experienced a sense of responsibility”.

**Table 3:** Students’ feedback regarding lecture and TBL as instructional strategies

Item		Response Emergency Module (*n* = 37)	Response Endocrinology Module (*n* = 35)
Overall course evaluation (Overall scale = 1–6)		4.8	5
What do you think was most interesting in the interactive lecture?	Interaction	16	15
	Content	6	7
	Teacher’s attitude	9	8
	Videos	5	3
	Others	1	2
Suggestions for improvement in lecture	More interaction	13	13
	More clinical examples	10	9
	More videos	5	5
	Reduce content	6	6
	Others	3	2
Should Interactive lectures be used as an instructional strategy in the future? Yes/ No response	Yes	31	30
	No	6	5
Experience of the interactive lecture as an instructional strategy (Overall scale = 1–6)		4.1	4.6
Experience of TBL as an instructional strategy (Overall scale = 1–6)		5.2	5.1
What do you think was most interesting in TBL?	Advance assignment	3	3
	Readiness Assurance Test (RAT)	3	5
	Team Application (tAPP)	21	17
	Peer feedback	8	9
	Others	2	1
Suggestions for improvement in TBL	Increase time for advance assignment	9	4
	Increase time for readiness assurance test	3	6
	Increase time for Instructor Clarification Review	2	5
	Increase time for Team Application	19	17
	Others	4	3
Should TBL be used as an instructional strategy in the future? Yes/ No response	Yes	31	29
	No	6	6
Which strategy, interactive lecture or TBL, is better for active learning?	IL	11	10
	TBL	26	25
How much percentage of a course should include interactive lectures as an instructional strategy? (From 0 to 100%)	100%	10	4
	75%	11	8
	50%	8	15
	25%	5	6
	None	3	2
How much percentage of a course should include TBL as an instructional strategy: (From 0 to 100%)	100%	8	3
	75%	9	7
	50%	13	17
	25%	4	6
	None	3	2
Which strategy, interactive lecture or TBL, do you think is better to achieve learning outcomes?	IL	19	15
	TBL	18	20
Which strategy, interactive lecture or TBL, do you think is better for clinical reasoning skills?	IL	10	11
	TBL	27	24

## Discussion

The students achieved the learning outcomes using both instructional strategies in our study, as more than 70% of students answered questions correctly in both modules. However, they attained higher marks with TBL as an instructional strategy (Table1& 2). Students scored higher with TBL as an instructional strategy in both modules, but it was statistically significant in groups 1 and 2 of the emergency module and group 1 of the endocrinology module (Table1). The results are variable in previous studies. In a few studies, TBL was found to be better in achieving outcomes [19, 20]. In another study, students were found to perform better in recall questions with the lecture as an instructional strategy, while, the results were comparable for applying knowledge [21]. A previous study found no significant difference in grades when comparing lectures with TBL if the variable of attendance is constant [22]. A recent study revealed that high achievers performed well in TBL and low achievers in IL even though there is no difference in students' overall performance using IL and TBL [23]. In another study, TBL was found to be superior in retaining knowledge compared with lecture as an instructional strategy. Students were also more satisfied and more engaged with TBL [24]. Another recent study noted that TBL was significantly superior to lecture in achieving higher grades [25]. One reason for the differences might be the inclusion of traditional lectures in some studies. The level of interaction matters and how an instructional strategy is being used; however, in most of the aforementioned studies, students have a higher level of satisfaction with TBL.

Another interesting parameter of our study was to compare TBL and IL in the learning of clinical reasoning skills. TBL was better in both groups of sixth-year students and group 1 of third-year students, while the difference was not significant in group 2 of third-year students. This finding assumes considerable significance in our context. Even though assessing this skill is not an easy job, clinical reasoning skill is mandatory in medical education curriculum [26]. We used MCQs to assess clinical reasoning. This is a widely used method, among other assessment strategies, to assess clinical reasoning [27]. Feasibility, internal consistency, and students’ awareness and comfort were the factors considered to use this method in our study. According to the literature, TBL is a better strategy to teach clinical reasoning skills [28–30]. TBL is superior in achieving higher-order outcomes and solving complex problems, and students prefer TBL over IL, and they found it better to work as a team [31]. So, based on our findings and literature support, we can safely recommend that TBL can be used to learn clinical reasoning skills in the undergraduate medical curriculum.

Students preferred interaction in lectures, among other factors, and they suggested increasing more interaction during lectures. In TBL, they preferred team application, suggesting a preference for involvement in the learning process (Table3). For a long time, it has been emphasized that active learning is better for students in most disciplines [32].

Students prefer TBL over IL in our study, as reflected by their feedback (Table3). TBL, as an instructional strategy, is preferred over other approaches due to many factors. TBL fosters students’ internal motivation, perceived learning, and autonomy, among other variables [33]. Even TBL is found superior to PBL by a cohort of students [34]. It has been observed that TBL enhances knowledge scores when compared to other methodologies, while participants' reactions are mixed [35]. As promoted by the US Department of Education Fund, TBL has been used in US and international schools for a long time [36]. It has been encouraged to use TBL as an instructional strategy due to different factors, such as the pressure of accreditation bodies to include active learning strategies, feasibility, cost-effectiveness, etc. However, the process of TBL assumes significance for better results that have an advance assignment, iRAT, tRAT, instructor clarification, tAPP, appeal if applicable, and peer feedback. TBL is also important to develop teamwork, communication skills, and collaboration to achieve academic (educational) outcomes [18]. A physician's skills or competencies are required, as mentioned in the SaudiMEDs framework [11]. Some competencies, for instance, teamwork and communication skills, were also evaluated in the modules by checklist, peer feedback, but these parameters were excluded in this study, so these factors are not part of the discussion.

We propose that it is important to incorporate more active learning strategies, such as TBL, in our curriculum because these strategies help students gain an in-depth understanding of the subjects.

Our study has some strengths. A randomized crossover design was used to compare both strategies, which is why every student was a part of each strategy, IL or TBL, at some stage. Secondly, both preclinical and clinical modules were selected. Both direct, in the form of quizzes, and indirect, evaluations were performed in the form of feedback.

However, our study was not immune to limitations. Firstly, this study is confined to two modules, so it cannot be generalized. The study was conducted in all-male campus somewhat further limiting its generalizability. The study was carried out in a medical college with an integrated modular system, which implies that the results might be different from colleges with a traditional curriculum. However, it can be used in similar contexts. Another issue is with regard to assessment; MCQ was used as an assessment method to assess both the outcomes and clinical reasoning skills. Although this method is supported by literature, and all efforts were made to construct good quality questions, we still view it as a limitation because the assessment of clinical reasoning skills is an onerous task [26]. Although students' feedback was obtained, in-depth perceptions of students are missing.

Another issue was that the in-class duration of IL was shorter than the TBL duration due to administrative constraints and the context of both strategies. This effect was mitigated by providing additional time to students for self-study in the library under direct supervision.

## Conclusion

Our study shows that students achieved LOs and CRS better with TBL as an instructional strategy. They preferred TBL over IL. It is suggested to include TBL, or increase its percentage, in the curriculum. Since they have an affinity for team application and feedback, TBL can be included in the curriculum, keeping in view the importance of clinical reasoning skills as a mandatory competency. Additional efforts should be undertaken to make lectures more interactive. Similarly, planning to conduct a TBL session is an important factor for better implementation. Further studies are suggested to determine the TBL outcomes over time, such as a semester, with well-formed and functional teams working together.

## Data Availability

All original data are available in the medical education unit, faculty of medicine in Rabigh, King Abdulaziz University, Jeddah, Saudi Arabia. The datasets collected and analysed during this study are available from the corresponding author on appropriate request.

## Abbreviations

IL: Interactive lecture
TBL: Team-based learning
CRS: Clinical reasoning skills
LO: Learning outcomes
CRQ: Clinical reasoning questions
CBE: Competency-based education
CBME: Competency-based medical education
PBL: Problem-based learning
CBL: Case-based learning
Saudi-MED: Saudi Medical Education Directives
FMR: Faculty of medicine in Rabigh
KAU: King Abdulaziz University
iRAT: Individual readiness assurance test
tRAT: Team readiness assurance test
MCQs: Multiple-choice questions
SBA: Single best answer
P: Difficulty index
DI: Discrimination index
